# A Model Analysis of Arterial Oxygen Desaturation during Apnea in Preterm Infants

**DOI:** 10.1371/journal.pcbi.1000588

**Published:** 2009-12-04

**Authors:** Scott A. Sands, Bradley A. Edwards, Vanessa J. Kelly, Malcolm R. Davidson, Malcolm H. Wilkinson, Philip J. Berger

**Affiliations:** 1 Ritchie Centre for Baby Health Research, Monash Institute of Medical Research, Monash University, Victoria, Australia; 2 Department of Chemical and Biomolecular Engineering, Faculty of Engineering, University of Melbourne, Victoria, Australia; University of California, San Diego, United States of America

## Abstract

Rapid arterial O_2_ desaturation during apnea in the preterm infant has obvious clinical implications but to date no adequate explanation for why it exists. Understanding the factors influencing the rate of arterial O_2_ desaturation during apnea (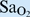) is complicated by the non-linear O_2_ dissociation curve, falling pulmonary O_2_ uptake, and by the fact that O_2_ desaturation is biphasic, exhibiting a rapid phase (stage 1) followed by a slower phase when severe desaturation develops (stage 2). Using a mathematical model incorporating pulmonary uptake dynamics, we found that elevated metabolic O_2_ consumption accelerates 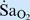 throughout the entire desaturation process. By contrast, the remaining factors have a restricted temporal influence: low pre-apneic alveolar 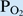 causes an early onset of desaturation, but thereafter has little impact; reduced lung volume, hemoglobin content or cardiac output, accelerates 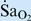 during stage 1, and finally, total blood O_2_ capacity (blood volume and hemoglobin content) alone determines 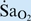 during stage 2. Preterm infants with elevated metabolic rate, respiratory depression, low lung volume, impaired cardiac reserve, anemia, or hypovolemia, are at risk for rapid and profound apneic hypoxemia. Our insights provide a basic physiological framework that may guide clinical interpretation and design of interventions for preventing sudden apneic hypoxemia.

## Author Summary

When breathing stops, the flow of O_2_ into and the flow of CO_2_ out of the body cease. Such an event, termed an apnea, can be especially dangerous in preterm infants in whom it can lead to a rapid decline in arterial O_2_ saturation, reaching rates of 3–8% per second, rapidly reducing O_2_ to a level that could lead to neurological damage. Despite extensive experimental research, we have a poor mechanistic understanding of the causes of rapidly developing hypoxemia. We describe a new mathematical model that allows examination of the importance of the major cardiorespiratory factors that are likely to influence the speed at which arterial hypoxemia worsens during apnea. We found that high metabolic rate as well as reduced pre-apneic ventilation, lung volume, cardiac output, hemoglobin content, blood O_2_ affinity, and blood volume accelerate the development of hypoxemia during apnea. Importantly, the cardiorespiratory factors that contribute to rapid hypoxemia are all pertinent to the preterm infant during early postnatal development. Thus the newborn is highly susceptible to rapid and severe desaturation, potentially explaining the propensity of preterm infants, particularly those with apnea, to neurological impairment.

## Introduction

Apnea and its accompanying arterial O_2_ desaturation are common clinical complications in preterm infants, occurring in more than 50% of very low birth weight infants [1]. In preterm infants, apnea causes a reduction in heart rate [2] and cerebral perfusion [3], often requires mechanical ventilation, and is associated with neurodevelopmental impairment [4]. Apnea-related hypoxemia is of major concern in light of evidence that repetitive hypoxia in newborn animals results in irreversibly-altered carotid body function [5], raising the possibility of impaired ventilatory control, and causes neurocognitive and behavioural deficits [6]. Respiratory arrest and hypoxemia are also strongly implicated in sudden infant death syndrome (SIDS) [7],[8] where the speed at which hypoxemia develops is considered to be particularly dangerous.

In preterm infants, the rate of arterial O_2_ desaturation (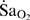) can be highly variable and rapid, with average rates as high as 4.3% s^−1^ during isolated apneas [9]. An earlier framework to describe 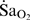 proposed that metabolic O_2_ consumption relative to alveolar volume determines the speed at which alveolar 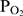 falls [10]; it was envisaged that 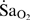 is then a function of falling 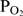 and the slope of the oxy-hemoglobin dissociation curve. However, such a model assumes that the rate of alveolar depletion of O_2_, denoted pulmonary O_2_ uptake (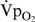), is equal to tissue O_2_ consumption during apnea (see Methods – Theory). Previous studies in adults have shown that 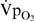 falls from metabolic consumption during apnea [11], and our previous modeling studies in lambs showed that the difference between 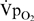 and metabolic O_2_ consumption has a crucial role in determining 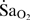 during recurrent apneas [12]. We found that apneic changes in 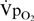 cause desaturation to occur in 2 stages. During stage 1, lung O_2_ stores are depleted, and 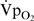 falls below metabolic consumption. During stage 2, 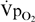 is close to zero, and tissue O_2_ needs are provided by depletion of blood O_2_ stores.

To date, no complete theoretical analysis of the factors influencing desaturation during apnea has been published. The only available study [13] has a number of critical limitations. First, the model incorporated a constraint of a fixed difference between 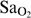 and mixed-venous saturation; thus dynamic changes in 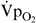 could not occur and their influence on 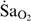 could not be examined. Second, no assessment was made of the impact of cardiorespiratory factors on the two stages of O_2_ desaturation. Third, in focusing on adults, the study did not examine profound desaturation to levels well below 60% as can often occur in preterm infants [9],[14].

Accordingly, the aim of the current study was to quantify the importance of cardiorespiratory factors relevant to 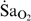 during apnea, with particular reference to the preterm infant. Using a model that permits variation of 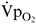 during apnea, we examine a number of factors known to influence 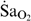, such as lung volume [15], metabolic O_2_ consumption [16] and pre-apneic arterial oxygenation [17] as well as factors that are particularly pertinent for the developing newborn, including anemia, hypovolemia, reduced O_2_ affinity, and chronically and acutely reduced cardiac output. We use the results to develop a conceptual framework for the interpretation of mechanisms underlying rapid 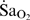 during apnea.

## Results

### Overview of the two-compartment model for gas exchange

**Figure 1:**
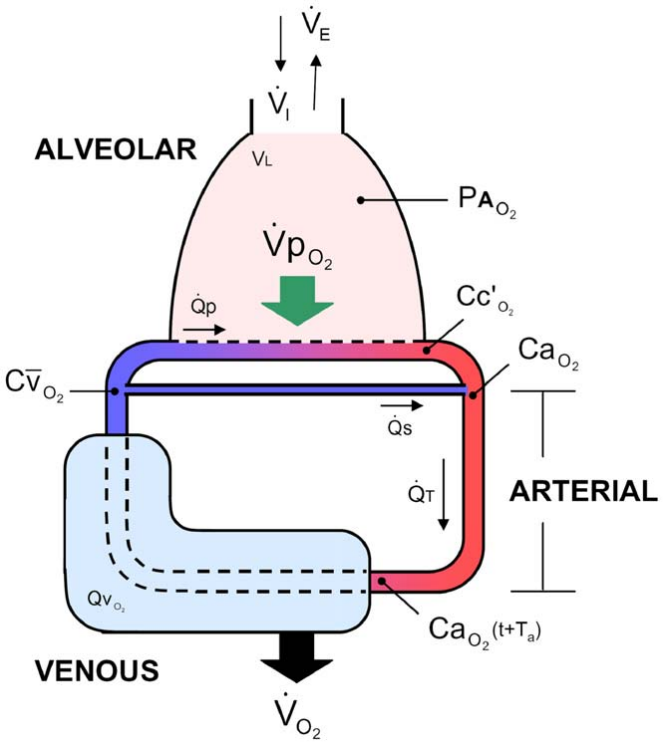
Model schematic representing O_2_ uptake, transport and consumption. O_2_ stores are represented by the alveolar, arterial, and venous compartments. Two dynamically-independent levels of O_2_ uptake are denoted: pulmonary O_2_ uptake (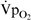) and metabolic consumption (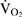). R-L shunt is also included. T_a_ is the arterial transit time. Symbols are described in Table 1.

To determine the independent influence of clinically relevant cardiorespiratory factors on 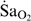 during a single isolated apnea, we used a two-compartment lung-body mathematical model which incorporated realistic blood O_2_ stores and gas exchange dynamics (Figure 1), as described in Methods – Mathematical model (a full list of symbols is provided in Table 1). We used published parameters for healthy preterm infants born at ∼30 wk gestational age (Table 2); the values represent measurements taken at approximately term equivalent age when surprisingly rapid desaturation has been observed [9]. We also derive analytic solutions for 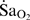 to quantify the importance of cardiorespiratory factors on 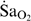 to obtain a detailed view of the arterial O_2_ desaturation process, as described in Methods – Theory.

**Table 1:** Mathematical symbols.

Symbol	Description
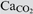	Arterial CO_2_ content
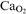	Arterial O_2_ content
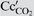	End-capillary arterial CO_2_ content
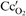	End-capillary arterial O_2_ content
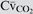	Mixed venous CO_2_ content
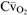	Mixed venous O_2_ content
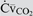	Rate of change in mixed venous CO_2_ content
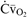	Rate of change in mixed venous O_2_ content
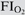	Fractional inspired O_2_
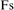	R-L pulmonary shunt fraction ( 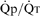 )
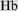	Hemoglobin content of blood
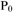	Barometric pressure, including conversion from STP to BTP, 863 mmHg
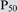	O_2_ partial pressure at 50% saturation
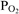	O_2_ partial pressure
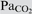	Arterial CO_2_ partial pressure
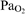	Arterial O_2_ partial pressure
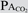	Alveolar CO_2_ partial pressure
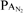	Alveolar N_2_ partial pressure
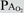	Alveolar O_2_ partial pressure
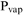	Alveolar water vapour partial pressure, 47 mmHg
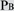	Barometric pressure, 760 mmHg
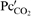	End-capillary CO_2_ partial pressure
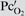	End-capillary O_2_ partial pressure
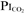	Inspired CO_2_ partial pressure
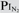	Inspired N_2_ partial pressure
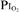	Inspired O_2_ partial pressure
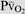	Mixed venous O_2_ partial pressure
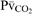	Mixed venous CO_2_ partial pressure
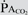	Rate of change in alveolar CO_2_ partial pressure
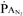	Rate of change in alveolar N_2_ partial pressure
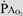	Rate of change in alveolar O_2_ partial pressure
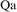	Arterial volume
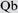	Blood volume
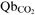	Blood volume for CO_2_
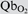	Blood volume for O_2_
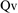	Venous (and tissue) volume
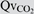	Venous (and tissue) volume for CO_2_
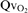	Venous (and tissue) volume for O_2_
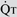	Cardiac output
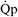	Pulmonary blood flow
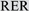	Respiratory exchange ratio ( 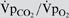 )
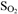	O_2_ saturation
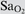	Arterial O_2_ saturation
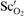	End-capillary arterial O_2_ saturation
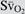	Mixed venous O_2_ saturation
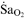	Rate of arterial O_2_ desaturation
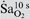	Average 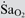 from t = 0–10 s during apnea
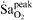	Peak instantaneous (‘linear’) 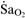 during apnea; stage 1
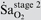	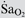 during stage 2
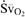	Rate of mixed-venous O_2_ desaturation
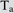	Arterial transit time
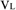	Lung volume
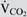	Metabolic CO_2_ production
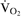	Metabolic O_2_ consumption
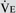	Expired alveolar ventilation
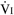	Inspired alveolar ventilation
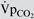	Pulmonary CO_2_ uptake (from capillary to alveoli)
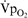	Pulmonary O_2_ uptake (from alveoli to capillary)
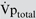	Net pulmonary gas uptake from alveoli to capillary
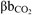	Capacitance co-efficient of blood for CO_2_
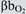	Capacitance co-efficient of blood for O_2_ relating changes in 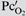 to changes in 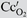
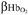	Capacitance co-efficient of hemoglobin for O_2_; slope of the O_2_-dissociation curve relating changes in 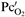 to changes in 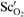

**Table 2:** Typical parameters for the preterm infant at term equivalent age.

Parameter	Value	Reference/s
Lung volume ( 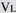 )	20 ml kg^−1^	[31],[54]
Metabolic O_2_ consumption ( 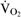 )	10 ml min^−1^ kg^−1^	[55],[39],[40]
Cardiac output ( 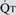 )	250 ml min^−1^ kg^−1^	[56]
Hemoglobin content (Hb)	8 g dl^−1^	[22]
P_50_	24 mmHg	[22]
Blood volume ( 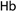 )	80 ml kg^−1^	[57]

P_50_ is the partial pressure at 50% saturation. 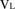 is taken from data on functional residual capacity. For all simulations unless otherwise stated: respiratory exchange ratio (RER) was assumed to be 0.8; shunt fraction (Fs) was adjusted to 8.7% to achieve a resting 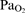 of 72 mmHg as is typical for normal healthy infants [58]; resting alveolar ventilation (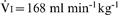 under normal conditions) was set to achieve resting 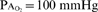.

### Pulmonary gas exchange dynamics during apnea

**Figure 2:**
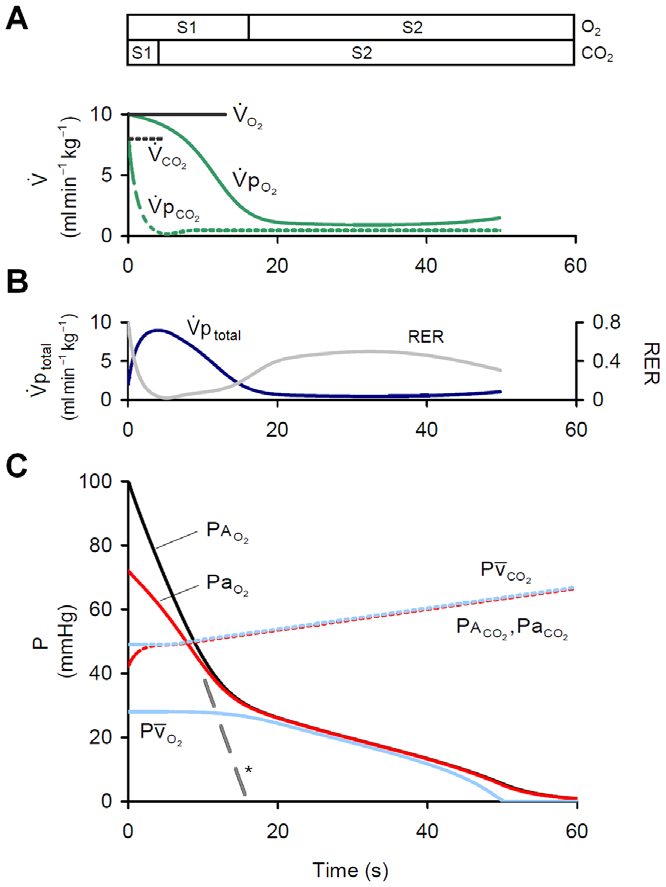
Pulmonary gas exchange during apnea. (A) Rate of pulmonary O_2_/CO_2_ exchange. 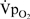 and 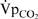 fall from resting levels during apnea. (B) Net alveolar-capillary gas uptake (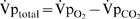) and respiratory exchange ratio (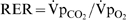)during apnea. (C) Changes in alveolar, arterial and mixed venous 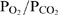 during apnea. Contrast the time-course in 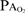 and 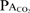 as they fall/rise towards 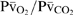. (*) represents the fall in 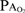 if 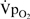 was assumed equal to 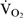. S1 = stage 1; S2 = stage 2.

To examine changes in O_2_/CO_2_ exchange during apnea, a single apnea was imposed on the model. During apnea, changes in alveolar O_2_ and CO_2_ stores are not constant (Figure 2); importantly, alveolar 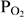 (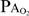) did not continue to fall at its initial rate as governed by metabolic O_2_ consumption (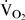), but instead the rate of fall in 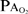 was reduced as it approached mixed venous 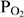 (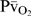), an observation also reflected in the falling 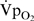. As a result, two distinct phases for O_2_ depletion can be seen, which we refer to as stage 1 and stage 2 [12]. During stage 1, 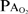 fell rapidly and 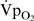 decreased and became dissociated from 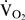; during stage 2, with 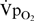 greatly reduced, both 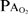 and 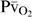 fell together at a reduced rate. The two distinct phases were also observed for alveolar and arterial 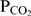 (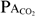, 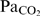) although stage 1 for CO_2_ was substantially shorter than that for O_2_. Such an effect results from the earlier fall in pulmonary CO_2_ uptake (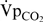) relative to the fall in 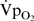 (Figure 2A) and is reflected in the reduction in respiratory exchange ratio (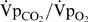) (Figure 2B). Consequently, a more rapid fall in 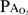 was observed compared with the rise in 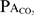
(see Methods – Derivation of equations), such that 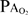 fell by 100 mmHg in the time 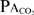 rose by just 14 mmHg (Figure 2C).

### Time-course of 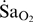 during apnea

**Figure 3:**
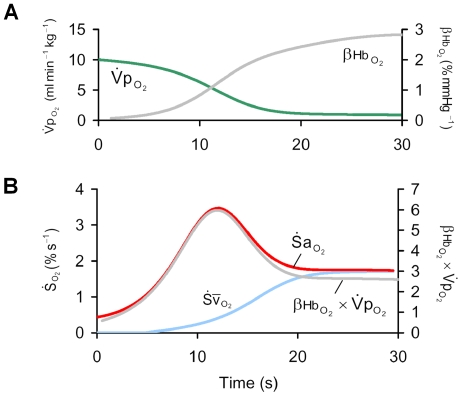
The time course of 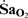 during apnea. Panel (A) shows the increase in the slope of the oxy-hemoglobin dissocation curve at the level of alveolar 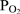 (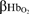), and the fall in pulmonary oxygen uptake (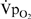) that occurs during apnea. Panel (B) shows that changes in the product 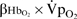 explain the time course of the instantaneous slope of arterial O_2_ desaturation (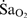) during apnea. Note that the peak 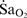 occurs when 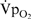 is substantially less than its resting value. Note also that the rate of fall of mixed-venous saturation (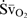) and 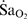 become equal and constant after 20 s.

The time-course of 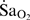 is complex (Figure 3), a consequence of the nonlinear O_2_-dissociation curve in combination with the fall in 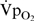. At apnea onset, 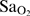 started to fall with a rate equivalent to that predicted by Equation 12, where 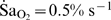 (Figure 3). During apnea, changes in the slope of the O_2_-dissociation curve (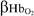) and 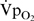 dominated the time-course of desaturation as hypoxemia progressed. As 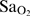 started to fall after apnea onset, 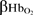 increased with little change in 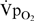, resulting in a proportional increase in 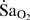. However, as arterial hypoxemia developed, there was a concurrent decline in 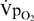. As 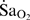 is directly proportional to the product 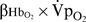 (Equation 11) it follows that during apnea, the peak 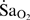 of 3.5% s^−1^ occurred when 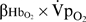 reached a maximum. This occurred when neither 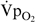 nor 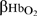 was at its maximum (both ∼50% of peak). Finally, with 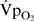 greatly reduced during stage 2, 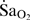 remained at a constant level (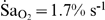), close to that predicted by Equation 13 (1.8% s^−1^).

### Factors influencing 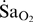


The following parameters were individually varied from their ‘normal’ values to quantify their influence on 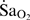: resting 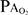, lung volume (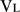), metabolic O_2_ consumption (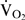), blood hemoglobin content (Hb), cardiac output (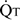), R-L shunt fraction (Fs), and the 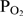 at 50% saturation (P_50_). All other parameters were kept constant to remove confounding effects, unless specified otherwise.

To quantify 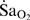 we used 3 different measures. First, since apnea is considered clinically significant if it lasts for >10 s and is accompanied by bradycardia or O_2_ desaturation [18], we calculated the average rate of fall in 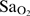 between apnea onset and 10 s later (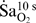); such a measure describes the immediacy of onset of desaturation and is analogous to the practical measurement of average 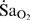 used in many clinical studies [9],[15],[19],[20]. Second, we determined the peak instantaneous 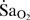 during apnea (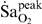), the value during the linear portion of arterial desaturation [10],[21] which we find is not confounded by resting 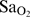. Third, we report a measure of 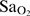 during stage 2 apnea (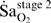). To quantify the sensitivity of 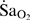 to changes in each cardiorespiratory factor, we defined the term impact ratio as the ratio of proportional increase in 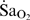 to a small increase from the normal value of each factor. For example, an impact ratio of 1 indicates a one-to-one increase in 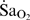 with an increase in the factor, and a negative ratio indicates an inverse relationship. The impact of each cardiorespiratory factor on 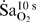, 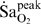, and 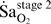 is summarised in Table 3.

**Table 3:** Impact ratios describing the effect of cardiorespiratory factors on 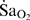.

Parameter alteration	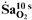	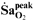	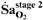
Resting 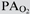	−3.97	−0.35	−0.01
Lung volume (VL)	−2.24	−0.82	−0.09
Blood volume (Qb)	−0.01	−0.06	−0.68
O_2_ consumption ( 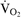 ) ^CC^	+2.29	+1.00	+1.00
O_2_ consumption ( 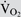 ) ^nCC^	+2.73	+1.92	+1.00
Hemoglobin content (Hb) ^nCC^	−0.38	−1.00	−0.89
Hemoglobin content (Hb) ^CC^	+0.01	−0.10	−0.89
P_50_	+1.37	−0.68	−0.11
Cardiac output ( 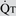 , resting)	−0.39	−0.90	0.00
Cardiac output ( 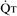 , transient)	+1.45	−0.06	0.00
Shunt Fraction (Fs)	−0.01	+0.01	0.00

Impact ratio is defined as the ratio of proportional increase in 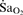 to the proportional increase in each factor, based on small changes around normal values. An impact ratio of 1 indicates a one-to-one increase in 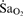 >with an increase in the factor, and a negative ratio indicates an inverse relationship. CC = cardiac compensated, nCC = cardiac uncompensated.

#### Resting 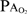


**Figure 4:**
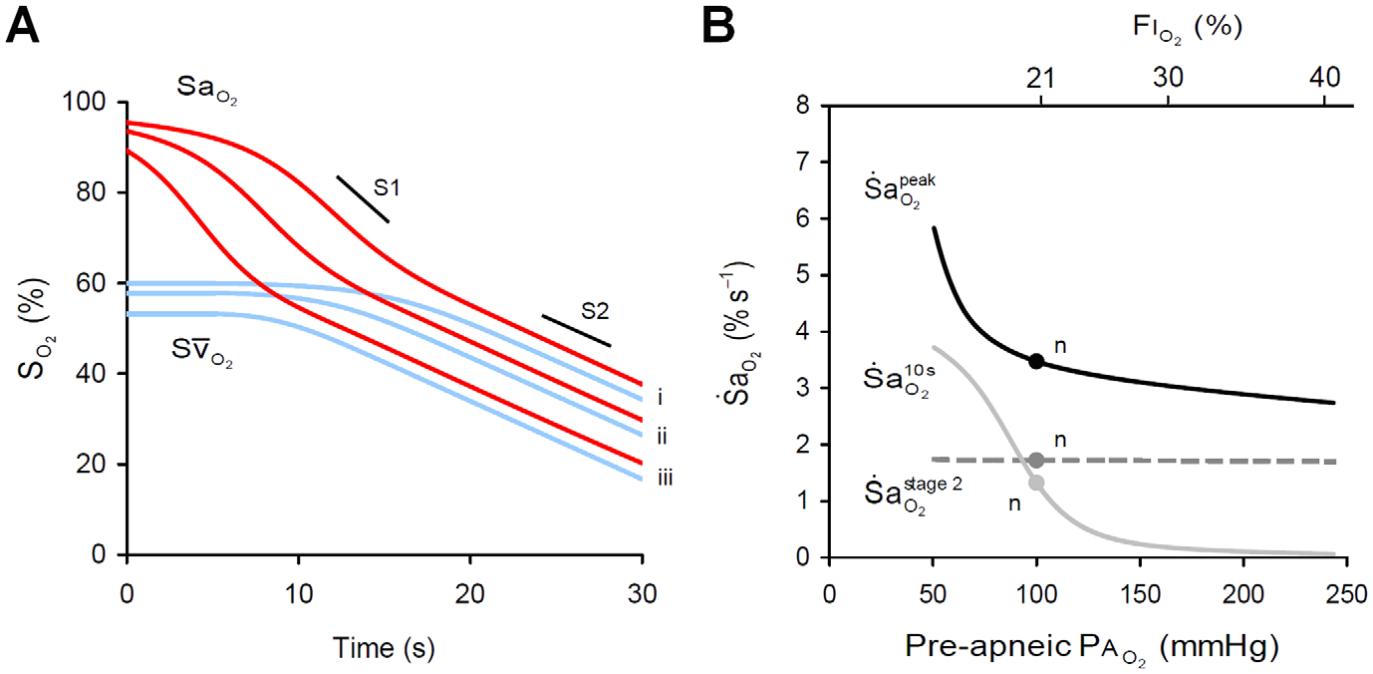
Impact of pre-apneic alveolar 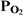 (ventilation, supplemental O_2_) on 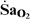. (A) Effect of three levels of alveolar 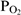
(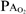), (i) 100 mmHg, (ii) 80 mmHg and (iii) 60 mmHg, on arterial (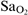) and mixed venous (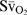) O_2_ desaturation during apnea. Note that arterial O_2_ desaturation is substantially right-shifted with increased 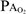. (B) Sensitivity of 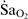 to changes in pre-apneic 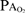
(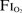). Note that reduced 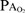 has a major impact on 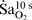 but little impact on 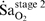; the influence on 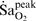 is small in the normal range but becomes stronger at low 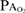. n = ‘normal’ 'values; S1, stage 1 slope; S2, stage 2 slope.

Changes in 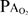, achieved via reduced resting ventilation or increasing inspired O_2_ (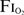), had a substantial effect on the onset of desaturation. Reduced pre-apneic 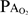 dramatically increased 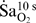 (Figure 4A), but had little effect on 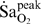 or 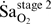. In contrast, increasing pre-apneic 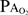 with the application of supplemental O_2_ achieved the opposite, essentially right-shifting or delaying the arterial desaturation curve, where one second of delay can be achieved by an increase in 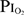 (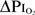) of ∼7 mmHg, or 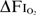 of ∼1% (see Methods – Derivation of equations). These results occurred despite only a minor influence being visible on resting 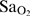. For example, a reduction of 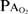 from 100 to 60 mmHg caused a 6% reduction in resting 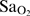 but at the same time led to a more than 2-fold elevation in 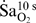 (Figure 4B). Additionally, a severe reduction in 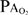, to below 70 mmHg, was required to elevate 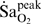.

#### Lung volume (VL) and blood volume (Qb)


**Figure 5:**
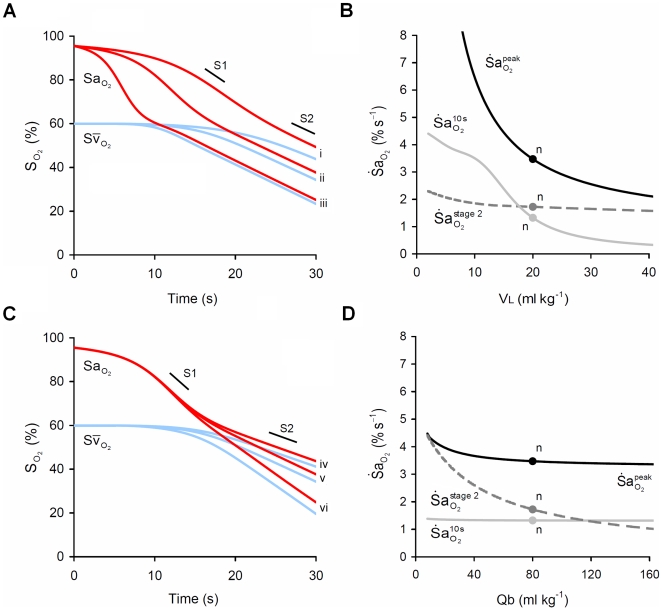
Impact of lung volume (VL) and blood volume (Qb) on 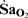. (A) Effect of three levels of VL, (i) 30, (ii) 20 and (iii) 10 ml kg^−1^, on arterial (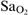) and mixed venous (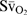) O_2_ desaturation during apnea. (B) Sensitivity of 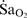 to changes in V_L_. Note that reduced V_L_ has a strong impact on 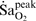 and 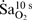 but no impact on 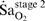. (C) Effect of three levels of Qb, (iv) 120, (v) 80 and (iv) 40 ml kg^−1^, on 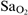 and 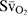 during apnea. (D) Sensitivity of 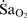 to changes in Qb. Note that reduced Qb has little impact on 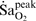 or 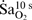 but has a large impact on 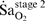. n = ‘normal’ values; S1, stage 1 slope; S2, stage 2 slope.

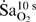
 and 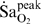 were inversely related to VL during stage 1 (Figure 5A, B), but changes in VL had no influence on 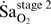. In direct contrast, reduced Qb strongly increased 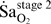, but had no effect on stage 1 desaturation as reflected in no change in 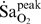 or 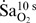 (Figure 5C, D).

#### Metabolic O_2_ consumption (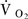)

**Figure 6:**
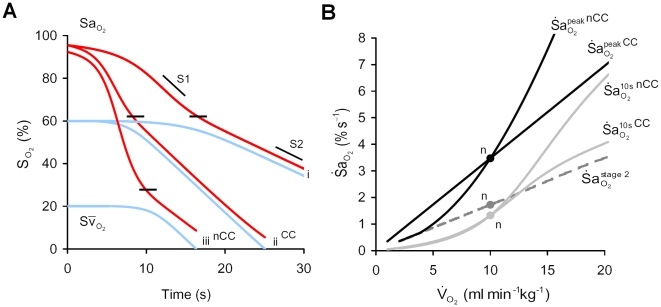
Impact of metabolic O_2_ consumption (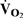) on 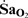. Panel (A) shows the effect of doubling 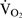 on arterial (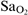) and mixed venous (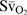) O_2_ during apnea; (i) 10 ml min^−1^kg^−1^, (ii) 20 ml min^−1^kg^−1^ with cardiac compensation (CC), and (iii) 20 ml min^−1^kg^−1^ with no CC (nCC). Note that with CC, increased 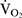, from (i) to (ii), elevated 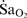 uniformly at all levels of 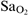 during both stages 1 and 2; note that the level of 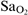 at the inflection point (shown by short black lines) is unchanged. With nCC (iii), increased 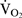 caused a reduced resting 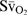 and lower 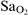 inflection, and greater 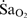 during stage 1, compared to (ii). (B) Sensitivity of 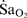 to changes in 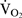. Note that with increased 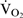: a uniform increase in 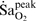 occurred with CC, and a more-than-proportional increase was seen with nCC; 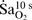 is elevated in both cases, but more so with nCC; a uniform increase in 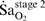 is shown regardless of CC. n = ‘normal’ values; S1, stage 1 slope; S2, stage 2 slope.

To examine the impact of changing 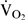 on 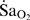, independent of resting 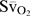, 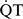 was adjusted to maintain resting 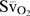 constant, where 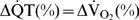; we refer to this procedure as ‘cardiac compensation’. Under this condition, elevated 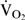 caused a directly proportional increase in 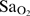 throughout stages 1 and 2 (Figure 6A, B). Without cardiac compensation, the effect of increased 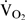 on 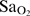 during stage 1 was magnified, as shown by the further increase in 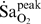 (Figure 6A, B).

#### Hemoglobin content (Hb) and oxygen affinity (P_50_)

**Figure 7:**
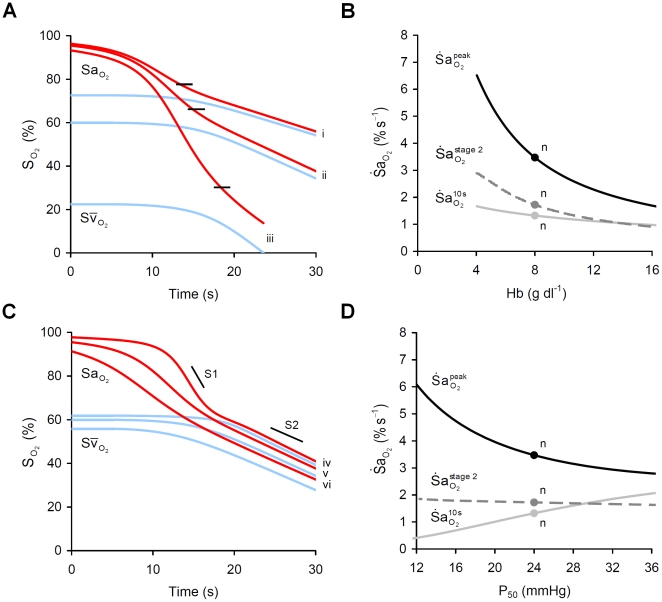
Impact of hemoglobin content (Hb) and O_2_ affinity (P_50_) on 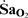. (A) Effect of three levels of Hb, (i) 12 g dl^−1^, (ii) 8 g dl^−1^ and (iii) 4 g dl^−1^, on arterial (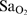) and mixed venous (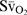) O_2_ desaturation during apnea. Note the fall in 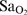 at the inflection point (shown by short black lines). Note also that the reduced Hb has little impact on desaturation above 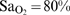. (B) Sensitivity of 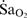 to changes in Hb. (C) Effect of three levels of P_50_, (iv) 18 mmHg, (v) 24 mmHg, and (vi) 36 mmHg, on 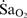. (D) Sensitivity of 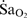 to changes in P_50_. n = ‘normal’ values; S1, stage 1 slope; S2, stage 2 slope.

Reduced hemoglobin content (Hb) increased 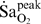 and 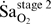 but had little effect on 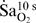 (Figure 7A, B). The increase in 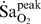 occurred with an increase in the peak of the product 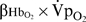 as 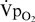 was higher at each level of 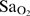. The simulation was repeated with cardiac compensation for the reduction in hemoglobin content, where 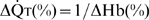, to maintain constant resting 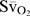. Following such compensation, no changes in 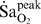 or 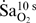 were observed but reduced Hb continued to increase 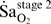. In examining the influence of P_50_, P_90_ was adjusted in equal proportion on the basis of published data [22]. Increased P_50_ increased the immediate 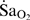, increased 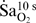, decreased 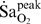 and had no effect on 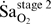 (Figure 7C, D).

#### Cardiac output (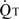)

**Figure 8:**
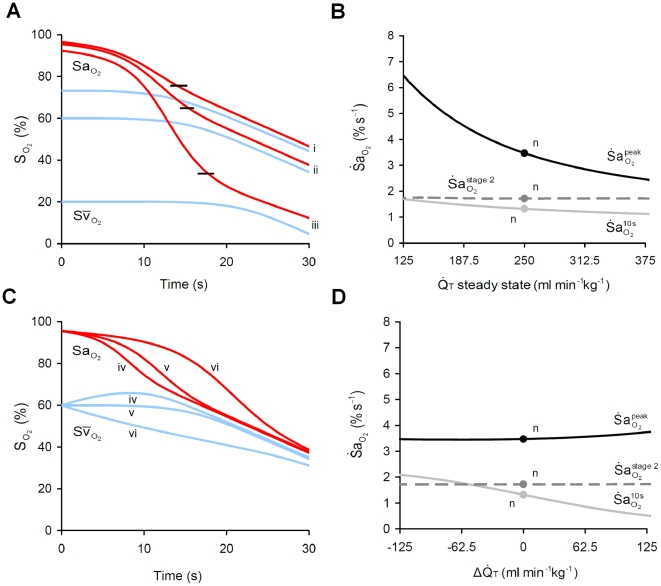
Impact of cardiac output (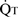) on 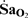. (A) Effect of three levels of resting 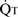, (i) 375 ml min^−1^kg^−1^, (ii) 250 ml min^−1^kg^−1^, and (iii) 125 ml min^−1^kg^−1^, on arterial (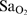) and mixed venous (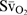) O_2_ during apnea. Note that reduced 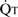 elevates 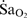, associated with a reduction in resting 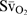 and reduction in 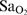 at the stage 1–2 transition or inflection point (shown by short black lines). (B) Sensitivity of 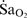 to changes in 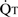. Note the strong influence of 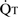 on 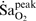, but negligible effect on 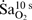 and 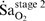. (C) Simulations in (A) repeated for a step change in 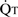 at apnea onset by (iv) +125 ml min^−1^kg^−1^ (e.g. tachycardia), (v) 0 ml min^−1^kg^−1^, and (vi) −125 ml min^−1^kg^−1^ (e.g. bradycardia), following resting 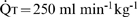. Note that the transient effect of 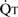 is opposite to the resting effect of 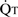 on arterial desaturation during apnea. (D) Sensitivity of 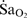 to acute changes in 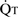 during apnea. Note the strong influence of a step-change in 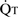 on 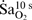, but negligible effect on 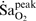 and 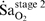. n = ‘normal’ values.

Reduced resting 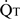 increased 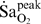, but had little impact on 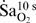 or 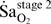 (Figure 8A, B). As with Hb, the increase in 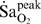 with reduced resting 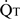 occurred with an increase in the peak of the product 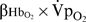. To differentiate between the influence on 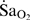 of an acute reduction in cardiac output, i.e. when bradycardia accompanies apnea, rather than a chronic reduction, we reduced cardiac output in a step-wise manner from the baseline value at the time of apnea onset. In constrast to the effect of reduced resting 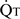, a transient reduction in 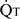 decreased 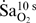, but had a negligible impact on 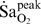 or 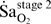 (Figure 8C, D).

#### Resting R-L shunt fraction (Fs)

**Figure 9:**
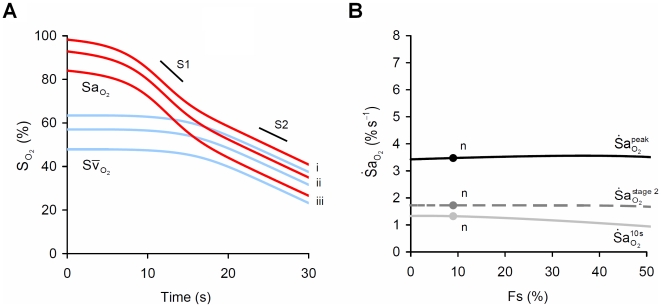
Impact of R-L shunt (Fs) on 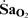. (A) Effect of three levels of Fs, (i) 0%, (ii) 15%, and (iii) 30%, on arterial (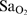) and mixed venous (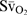) O_2_ during apnea. Note that resting R-L shunt fraction has a negligible impact on 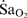 during apnea. (B) Sensitivity of 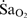 to changes in Fs. n = ‘normal’ values; S1, stage 1 slope; S2, stage 2 slope.

Increased Fs reduced resting 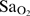 and 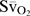 but had no effect on 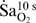, 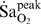, or 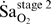 (Figure 9A, B).

## Discussion

Our model analysis of the rate of arterial O_2_ desaturation during apnea demonstrates that pre-apneic ventilation, lung volume, cardiac output, hemoglobin content and blood volume exert unique effects on 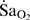 throughout the time-course of desaturation, while metabolic O_2_ consumption is uniformly influential throughout the process. Our analysis reveals that lung volume and the slope of the O_2_-dissociation curve are important early in the process, during what we refer to as stage 1 [12], but not stage 2. For the first time, our study reveals that reduced cardiac output and hemoglobin content, and as a consequence resting mixed-venous saturation, substantially accelerate peak 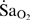. Finally, low blood volume and hemoglobin content, and therefore a low total blood O_2_ capacity, increase the speed of desaturation, but only in stage 2. In addition to infants with elevated metabolic needs and low lung volume, those with anemia, cardiac dysfunction, or hypovolemia, which are common complications of prematurity, are at heightened risk of rapid and profound arterial desaturation during apnea.

### Methodological considerations

To evaluate the independent effects of cardiorespiratory factors on 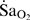 we used a two-compartment model, incorporating both alveolar and blood gas stores. The inclusion of a realistic blood store was crucial to reveal that changes in 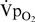 occur as a consequence of arterial and mixed-venous saturation falling asynchronously during apnea (Figure 3). Our approach allowed us to extend the previous framework based on the assumption of constant 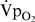 [23], which prevented the recognition that a steep O_2_-dissociation curve and low lung volume do not accelerate 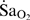 beyond stage 1. Furthermore, the varying 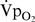 permitted recognition that cardiac output, hemoglobin content, and blood volume have a major influence on 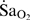.

In the current study, the typical value of 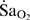 found using our model was 3.5% s^−1^ whereas Poets and Southall [9] using beat-by-beat oximetry in preterm infants reported a mean value for 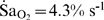 during isolated apneas. Reasons for our lower value may lie with our simplifying assumptions. Notably, we assumed a homogenous lung compartment and complete gas mixing and as such, the model incorporated neither limitation of alveolar-capillary diffusion nor an uneven ventilation-perfusion distribution, two factors that could cause an increase in 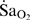. In addition, we assumed a constant lung volume during apnea, equal to published values of functional residual capacity, whereas it is known that lung volume can fall during apnea [15],[24]; based on our data, a fall in lung volume to 15.5 ml min^−1^kg^−1^ immediately after apnea onset would achieve 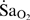 of 4.3% s^−1^ (Figure 5B).

A final assumption implicit in our model is that all O_2_ transfer to the blood occurs via the pulmonary circulation. However, in very preterm infants there is evidence of percutaneous respiration in the first few days of life in both room air and with supplemental O_2_ [25]. With whole body exposure of 90% O_2_ to the newborn skin, it has been calculated that 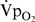 can be reduced by 8–10% [26], likely via an increased resting mixed-venous saturation; our study demonstrates that such an effect would decrease 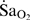 during apnea.

### Pulmonary gas exchange dynamics during apnea

Our study is consistent with previous observations that 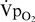 and 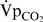 rapidly decline during apnea from their steady-state values [11], with 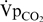 falling faster than 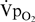. The relatively low blood capacitance for O_2_ compared with that for CO_2_ results in the resting alveolar–mixed-venous partial pressure difference being ∼12-fold greater for O_2_ than for CO_2_. Consequently, when apnea begins ∼12 times more O_2_ than CO_2_ must diffuse across the lung to obliterate the alveolar–mixed-venous partial pressure difference. The slower fall in 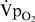 vs. 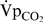 provides for a faster depletion of alveolar O_2_ vs. CO_2_ stores; such an effect results in complete desaturation of arterial blood in the time 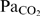 rises by just 14 mmHg. These findings lead us to conclude that short-term O_2_ homeostasis is more unstable than CO_2_ homeostasis and thus that the danger of isolated apneas in infants is likely to be mediated via hypoxemia rather than hypercapnia.

### Factors influencing 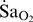


Our study provides for the first time a comprehensive analysis of the factors that determine arterial desaturation during apnea in preterm infants. We show that resting oxygenation in the form of alveolar 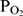 has the greatest influence on desaturation at apnea onset. When apnea begins at an increasingly lower alveolar 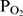, 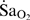 more quickly reaches its maximum because 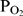 rapidly arrives at the steepest part of the O_2_-dissociation curve. This effect explains the inverse relationship between mean 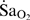 and pre-apneic 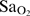 during apnea [17], but as we show the peak slope itself is negligibly affected by reduced resting 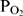 within the normal range.

We demonstrate that 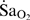 is inversely related to lung volume during stage 1 of apnea as a result of the greater reduction in alveolar 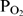 in poorly inflated lungs per unit of O_2_ transferred into the pulmonary capillaries. This analysis is consistent with the inverse correlation between 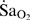 and lung volume [15], with the view that active upper airway closure maintains lung volume and slows 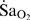 [27],[28], and with our recent report that the application of continuous positive airway pressure effectively slows 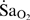 in lambs [29]. However, once stage 2 begins, the blood becomes the principal source of O_2_ and thus the only store which influences 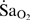.

A novel finding from our study is that reduced resting mixed-venous saturation, caused by either a reduced cardiac output or reduced hemoglobin content, strongly elevates peak 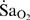, independent of metabolic O_2_ consumption. We show that reduced resting mixed-venous saturation accelerates 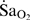 via an increase in the peak value of 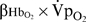; in other words, low mixed-venous saturation provides for a greater pulmonary O_2_ uptake even in the presence of a developing arterial hypoxemia, and thereby increases 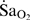. A role for hemoglobin in determining 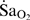 is consistent with the finding that elevated hemoglobin content in adults slows 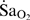 during apnea [21]. In contrast, blood transfusion to raise hemoglobin content in anemic preterm infants, a common clinical therapy, has little or no impact on the severity of apneic desaturation [30]. Our proposed explanation for the lack of benefit of raising hemoglobin content via transfusion is that it also reduces heart rate [30] and cardiac output. Thus, in the newborn, the rise in mixed-venous saturation expected after transfusion is counteracted by a tendency for mixed-venous saturation to fall as a result of reduced cardiac output. An investigation that failed to find an effect of cardiac output on 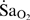 [23] did not account for our finding that pre-apneic and transient changes in cardiac output have opposing influence on 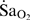. Importantly, we find that a transient fall in cardiac output, characteristic of bradycardia during apnea in preterm infants [2], conserves alveolar O_2_ via reduced 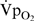 and thus reduces 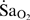 (see Equations 10 and 11). Consistent with this finding, apneic bradycardia prevents a rapid fall in 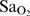 in adults [21].

**Figure 10:**
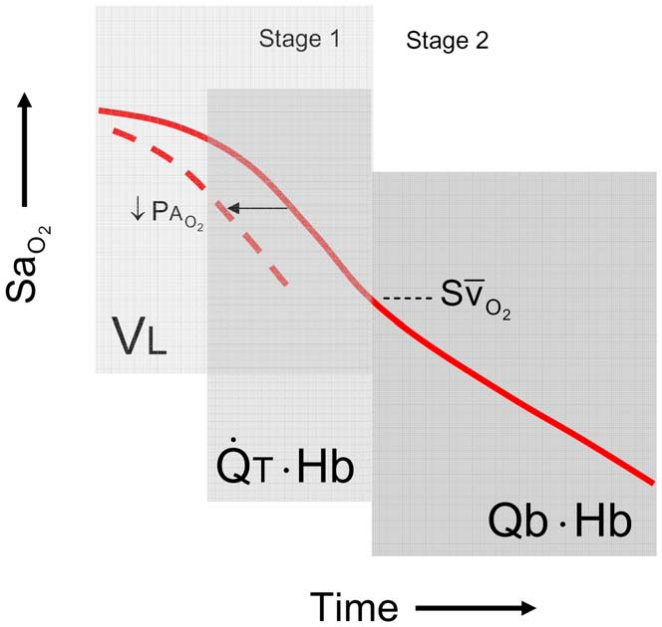
Conceptual framework depicting the temporal sequence of influence of the key cardiorespiratory factors on 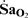. Note the regions of influence of lung volume (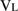), cardiac output (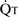) and blood volume (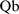), each with respect to metabolic O_2_ consumption (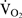). Hemoglobin content (Hb) influences the latter phase of stage 1 as well as stage 2. The impact of reduced 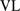 is limited to stage 1, and blood volume to stage 2. Reduced 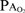 causes a leftward shift in the desaturation trajectory. Note that the point of inflection at the transition between stages reveals the resting 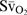.

We found that each of the factors examined exerts a unique and therefore recognisable influence on the time course of the desaturation process (Figure 10). Low alveolar 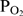 can be recognised by a left-shift of the desaturation trajectory so that desaturation begins sooner following the onset of apnea. A steep desaturation slope in the early phase of stage 1 points to a low ratio of lung volume to metabolic O_2_ consumption. In the late phase of stage 1, when desaturation proceeds in a linear fashion, a low resting mixed-venous saturation accelerates 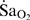 and leaves the fingerprint of a low inflection point in arterial O_2_ desaturation; low resting mixed-venous saturation reflects low cardiac output or hemoglobin content with respect to O_2_ consumption. Lastly rapid 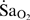 during stage 2 signifies a low total blood O_2_ capacity with respect to O_2_ consumption which would point to either low blood volume or anemia. The presence of a constant R-L shunt, while having no influence on 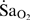, causes a parallel downwards shift in the desaturation trajectory. The unique impact of different factors on the desaturation curve may be used to guide preventive clinical intervention.

### Clinical significance

We show theoretically that the lower lung volume [31] and higher metabolic O_2_ consumption [32] of preterm compared to term infants predisposes to a rapid onset and progression of desaturation during apnea. Two reports offer support for this view. First, rapid desaturation occurs in infants with low functional residual capacity [15], a finding that may help to explain the more frequent O_2_ desaturation events during active sleep [33] when functional residual capacity is reduced. Second, frequent desaturation is characteristic of preterm infants with bronchopulmonary dysplasia (BPD) [34] whose O_2_ consumption is 25% greater [35], and functional residual capacity is 25% less [36], than in preterm infants without BPD; Equations 11 and 12 predict that such differences increase both immediate and peak 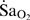 by ∼70%. In addition, hypoventilation and reduced resting 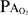 in infants with BPD, as inferred from elevated 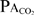 [37], further increase desaturation at apnea onset. Our finding that each rise of 1% in inspired O_2_ provides ∼1 s of delay (right-shift) in the onset of apneic desaturation (Equation 15) may guide the titration of supplemental O_2_ for the prevention of apneic hypoxemia while minimising the well known side-effects of long-term exposure to hyperoxia.

Our study has implications for the management of infants in clinical care. Metabolic O_2_ consumption can be elevated after feeding [38], with reduced ambient temperature [39], and via the adminstration of methylxanthines [40]. Despite the success of methylxanthines in reducing the frequency of apnea and bradycardia, such treatment has surprisingly little impact on hypoxemic episodes [41]; we suggest that the elevated O_2_ consumption and the absence of bradycardia are likely to increase 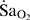 during those apneas that persist despite treatment. The severity of hypoxemic episodes is reduced by switching preterm infants from supine to prone [42], which may increase functional residual capacity [43] and improve diaphragm function, increase tidal volume and increase resting alveolar 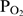 [44]. Our finding that low cardiac output leads to increased 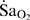 during apnea leads to the suggestion that judicious adjustment of inotropic support in infants with cardiac abnormalities could improve resting mixed-venous saturation and reduce apneic hypoxemia.

Hypoxemic events become less frequent between infancy and childhood, despite an unchanged apnea frequency [28], perhaps as a result of a fall in O_2_ consumption per body weight. However, before this occurs, infants experience a period of susceptibility to rapid desaturation during apnea as a result of a fall in hemoglobin content and O_2_ affinity [22] and a rise in O_2_ consumption [45]. The implications for SIDS are obvious in that these changes coincide with the peak incidence for SIDS at 2–3 months [46]. SIDS also occurs disproportionately in preterm infants [46], who manifest severe anemia [22] and greater O_2_ consumption. Infants resuscitated from apparent life threatening events have been found to have lower hemoglobin content [47], pointing to a potential role for rapid 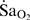 in the progression of such events. It is possible that the rapid development of apneic hypoxemia initiates prolonged hypoxic cardiorespiratory depression that in turn leads to SIDS.

### Conclusion

We have provided a mathematical framework for quantifying the relative importance of key cardiorespiratory factors on the rate of arterial O_2_ desaturation during apnea, with particular relevance to preterm infants. For the first time we have demonstrated that each of the factors examined has a signature influence on the trajectory of desaturation, providing quantitative insight into the causes of rapidly developing hypoxemia during apnea.

## Methods

### Mathematical model

#### Lung compartment

For the lung, a single homogeneous compartment is assumed based on the model of Grodins et al [48]. Each equation describing changes in the alveolar partial pressure of each gas (G) is based on the conservation of mass (specifically, the pressure–volume product) and is expressed in terms of inspired and expired alveolar ventilation and transfer of gases into the pulmonary capillary:
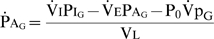
(1)where 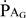 represents the rate of change of alveolar 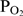, 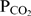, and 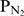; 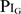 represents the inspired alveolar partial pressure of each gas G; P_0_ is atmospheric pressure converted from STP to BTP (863 mmHg); 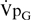 represents 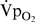 and 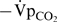, pulmonary gas uptake (STPD) for O_2_ and CO_2_ (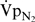 was neglected in this study for simplicity); 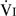 and 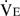 are inspired and expired alveolar ventilation (BTPS). Accounting for the difference in 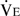 and 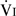 due to a net pulmonary gas uptake into the pulmonary blood, yields:
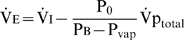
(2)where 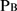 = barometric pressure (760 mmHg); 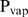 = water vapour pressure (47 mmHg); 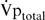 is the net pulmonary gas uptake, 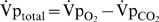.

Since purely obstructive apneas are relatively rare in preterm infants [49], an unobstructed airway was chosen as the standard model in this study. In the current study it was assumed that lung volume did not fall during apnea, as in active sleep [24], when apneic desaturation events are most common [33]. With lung volume constant, conservation of mass requires that passive airflow into the unobstructed airway must occur in response to a net pulmonary gas uptake into the pulmonary blood [11]. To account for this effect, we can write:
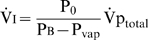
(3)Pilot simulations predicted that the volume of gas inflow during apnea is unlikely to exceed physiological deadspace. Thus, during apnea 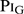 is taken as 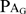 of the last exhaled breath prior to apnea onset.

For the current study we assumed diffusion equilibrium at the pulmonary capillaries, such that 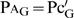. Gas uptake is determined from the Fick equation; specifically, pulmonary blood flow (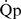), and the difference between end capillary (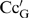) and mixed venous (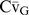) content:
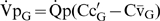
(4)Utilising equations for R-L shunt, arterial content of each gas G is determined from its end capillary (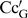) and mixed venous (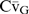) content, and pulmonary shunt fraction (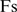):
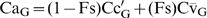
(5)Fs defines the ratio of pulmonary blood flow to cardiac output, such that Fs = 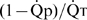.

#### Body compartment

Assuming that the 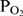 of the venous blood is equilibrated with the tissue 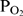, the mass-balance equations are:
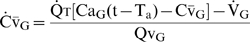
(6)where 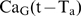 represents the gas content of O_2_ and CO_2_ in the arterioles; T_a_ is arterial transit time; 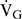 represents 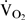 and 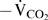, the metabolic consumption of O_2_ and production of CO_2_; 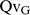 represents 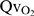 and 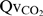 the combined venous/tissue volumes for O_2_ and CO_2_.

Blood O_2_ stores were partitioned by assigning blood volume (Qb) to arterial (25%) and venous (75%) compartments [50] and they were modelled assuming an entirely unmixed arterial compartment, and an entirely mixed and homogenous venous compartment. The arterial transit time (T_a_) is constrained by the arterial volume (Qa) by the relationship 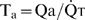. The body compartment volume 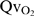 is taken as the venous volume. 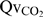, the effective venous/tissue volume for CO_2_ is taken as the same value for Qv_O_2__, based on published data (see Methods – Derivation of equations). Physiologically this represents no additional contribution of a specific tissue reservoir for CO_2_ within the time frame of apnea.

To characterise the O_2_-dissociation curve we used a modified form of the equation of Severinghaus [51]. We re-expressed the equation with respect to the partial pressure at 50% (P_50_) and at 90% (P_90_) saturation:
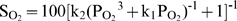
(7)where 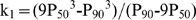 and 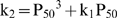. Values for P_50_ (24.0 mmHg) and P_90_ (53.6 mmHg), were obtained from the data of Delivoria-Papadopoulos [22] for a 9–10 wk-old preterm infant. O_2_ content (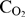, ml ml^−1^) includes that bound to hemoglobin (Hb, g ml^−1^) and that dissolved in plasma:
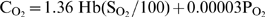
(8)The relationship between CO_2_ content (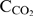) and 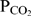 was assumed linear:
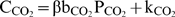
(9)where 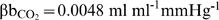 and 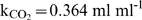 as adapted for STPD from Grodins et al. [52].

Simulations were performed using software written in MATLAB (The Mathworks; Natick, MA).

### Theory

#### A general equation

In an earlier study we developed a general relationship that describes the factors influencing the magnitude of 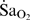 at any instant in time during apnea [12]:
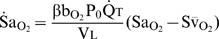
(10)where 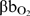 is the capacitance co-efficient of blood for O_2_. To specifically demonstrate the role of gas exchange, it is more useful to represent 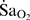 in terms of 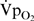. Using Equation 1 for O_2_ under conditions of apnea (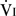,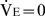), assuming 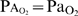, and using 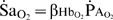, reveals:
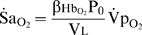
(11)where 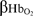 (% mmHg^−1^) is defined as the slope of the O_2_-dissociation curve, specifically regarding end-capillary 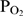 with respect to 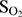. It is clear from Equation 11 that 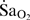 is directly proportional to the product 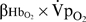, which both vary substantially during apneic arterial desaturation. Although Equations 10 and 11 are useful conceptually, values for (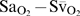) or 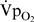 throughout apnea are unknown, and thus 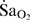 is not simple to predict explicitly.

#### Special cases

**Figure 11:**
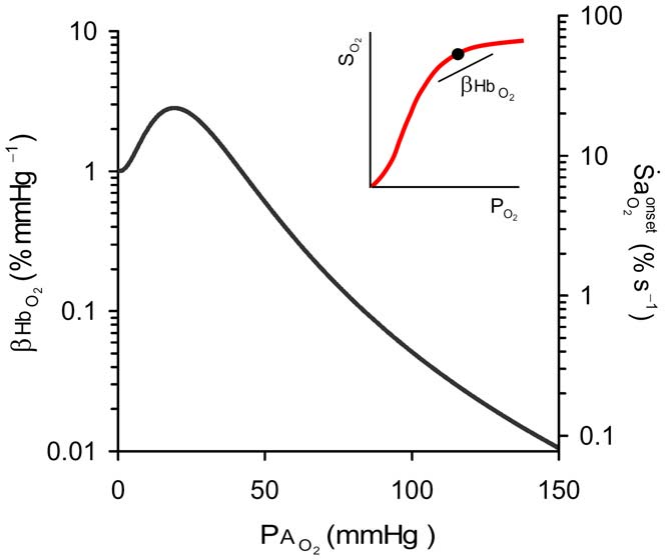
Relationship between the slope of the oxy-hemoglobin dissociation curve and alveolar 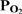. Note that reduced alveolar 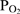 (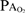) causes a substantial increase in the slope of the oxy-hemoglobin dissociation curve (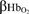; see inset) and in 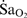 at apnea onset (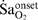; based on Equation 12).

The original framework to understand factors influencing 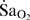 was based on the assumption that 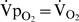 [10],[23] which does not hold true during apnea [11],[12]. However, such an assumption is valid prior to any substantial fall in 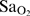, and as therefore useful to explicitly describe 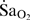 immediately upon apnea onset (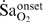):
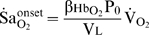
(12)Notably, Equation 12 demonstrates that for any level of 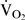 and 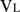, 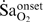 is intimately related to 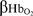. Consequently, 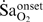 increases dramatically with reduced resting 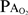 (Figure 11).

Although no simple expression could be written to describe 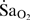 explicitly for stage 1, we derived an expression for 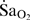 during stage 2 (see Methods – Derivation of equations), given by:
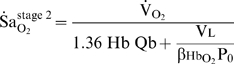
(13)Since the total blood O_2_ capacity (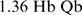) is much greater than 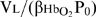, 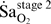 is determined principally by (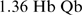) with negligible influence coming from lung volume (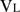) and the slope of the O_2_-dissociation curve (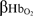), as well as 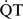. Using the values for the preterm infant in Table 2 and maximum 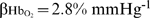, Equation 13 predicts that 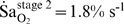. The little remaining 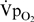 during stage 2 can be found by combining Equations 11 and 13:
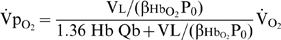
(14)Equation 14 predicts that 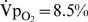 of its resting value during stage 2. Notably, 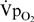 is increased by reducing Hb and Qb; the greater 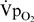 and thus a greater rate of alveolar O_2_ depletion with reduced blood O_2_ capacity (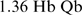) will increase 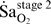.

How can we reconcile that Equation 13 shows that 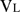 no longer influences 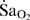 during stage 2, when the general equation (Equation 11) implies that reduced 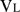 will accelerate 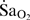 throughout apneic desaturation? Equation 14 reveals that during stage 2, elevated 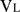 also acts to increase 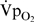; thus nearly entirely offsetting the direct influence on 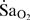. The same applies for reduced 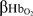, which acts to elevate 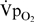 and therefore no longer accelerates 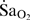 during stage 2.

### Derivation of equations

Here we derive the explicit equations used within the current study to encapsulate key relationships pertaining to gas exchange and arterial desaturation during apnea.

#### Stage 2 arterial O_2_ desaturation

This section details the derivation of an explicit equation to predict the rate of both arterial and venous desaturation during the severe desaturation of stage 2, a phase where 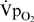 is substantially reduced below 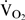 and both 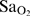 and 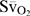 fall at the same rate. Ignoring dissolved plasma O_2_, consideration of Equation 1 for O_2_ and assuming 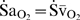 yields:
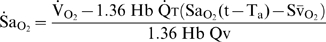
(15)


By substituting the following relationships into Equation 15: 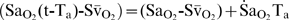; 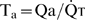; Qb = Qa+Qv; 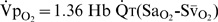; it can be shown that 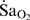 is directly proportional to the difference between 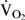 and 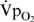, where:
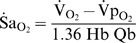
(16)Combining Equations 11 and 16 yields Equation 13.

#### Estimation of effective blood volume for CO_2_

Using the same methodology as described above, the ratio of 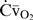 to 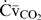 during stage 2 can be used to estimate the ratio of 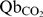 to 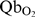. 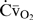 and 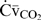 can be found using:
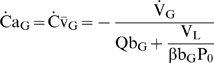
(17)where 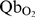 and 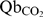 are the effective blood volumes for O_2_/CO_2_; 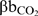 is the capacitance coefficient for CO_2_. Neglecting pulmonary gas exchange, combining Equation 17 for O_2_ and CO_2_ gives:
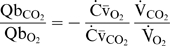
(18)Equation 18 permitted the calculation of 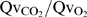 based on published data [53; their Figure 3] where during apnea the rate of rise in 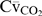 is very close to the rate of fall in the product of 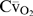 and the respiratory exchange ratio (RER); using 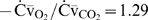 from their data, and assuming resting RER = 0.8, we find that 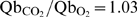 or approximately 1. Thus 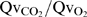 is assumed to be 1.

#### Stage 1 hypercapnia

Here we develop a relationship to describe the time-course of alveolar/arterial hypercapnia during stage 1 for CO_2_. Using Equation 1 for CO_2_, taking 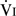,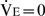, gives the relationship 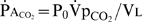. Substituting the steady-state Fick equation, 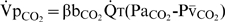, assuming alveolar-arterial equilibrium (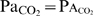), using 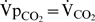 under resting conditions, assuming that 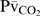 is constant, and solving for 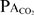 yields:
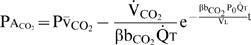
(19)Calculating the rate of rise in 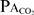 (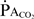) by taking the derivative gives:
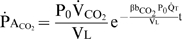
(20)


Equations 19 and 20 describe the slowing of 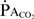 from the initial rate 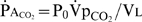 as 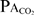 rises towards 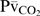. Specifically, the time constant 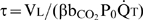 demonstrates that high 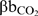 causes a rapid slowing of 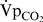 and hence of 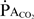 as the arterial value approaches venous value. Indeed, fitting an exponential curve to the 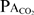 trace (Figure 2) during the first 5 s of apnea yielded a rapid time constant of 1.26 s, a value close to that predicted by 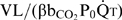. The corollary is that the low value of 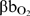 prevents the slowing of 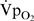 as desaturation progresses, giving rise to a rapid 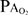 decline and thus rapid arterial desaturation. Likewise, further reducing 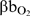 by lowering hemoglobin content potentiates such effect.

#### Impact of supplemental O_2_

The delay (right-shift) in arterial desaturation during apnea with increasing supplemental O_2_ (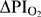) can be predicted explicitly. Using Equation 1 for O_2_ under the conditions of apnea, and assuming 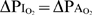, the delay (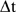) in arterial desaturation is given by:
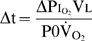
(21)

